# Survey Study of Awareness and Perception of Palliative and Hospice Care in a Cancer Center in Rural Pennsylvania

**DOI:** 10.1089/pmr.2020.0110

**Published:** 2022-02-21

**Authors:** Zhu Wang, Kathy Selvaggi, Dillon Stein

**Affiliations:** ^1^ Department of Family Medicine, Clarion Hospital, Butler Health System, Clarion, Pennsylvania, USA.; ^2^ Department of Palliative Care, Butler Memorial Hospital, Butler, Pennsylvania, USA.

**Keywords:** cancer center, oncology patient perspective, palliative and hospice care, rural community hospital

## Abstract

***Purpose:*** Hospice and palliative medicine (HPM) have been gaining ground especially in large urban settings. However, less is known about their perception in small rural areas. This study assessed the awareness and perception of a rural oncology population of this field and the effects of prognosis on their awareness.

***Methods:*** Subjects were patients of the community cancer center in rural Clarion County, Pennsylvania, who volunteered to complete a short nine-question survey (supplemental figure). Results were analyzed based on completed surveys.

***Results:*** A total of 65 surveys were collected from the Cancer Center at the Clarion Hospital. Among these patients, 54% stated that they have heard of palliative and hospice medicine. When correlating patient-reported prognosis with their awareness of palliative and hospice care, 100% of the patients with poor prognosis were aware of palliative or hospice care, respectively. In contrast, only <20% of patients with reported good prognosis were aware of HPM.

***Conclusions:*** Our study has shown that the awareness of HPM in rural areas is improving. It was observed that more patients are aware of HPM is when their prognosis was poor as compared with those who reported good prognosis.

## Introduction

Hospice and palliative medicine (HPM) is a field that supports patients with serious illness, including those at end of life. In the 1950s, studies of end-of-life care were performed and showed that there were large needs in patients with terminal illness^1^ and these care gaps persisted for decades.^2^ Even in 2018, a national study based on Google searches of “comfort care” showed that searches quadrupled in February 2018 after former First Lady, Barbara Bush, issued a statement that she would not receive additional medical treatments but “instead focus on comfort care.”^3^ Much research has been performed in large institutions, but few studies have focused on small rural hospitals. Some studies observed similar needs and concerns of patients in rural and urban settings^4,5^ whereas others have reported that rural populations are less informed.^6,7^ This study seeks to determine if the recent growth of the HPM field has resulted in a change of perception in a rural population.^8^

## Methods

The study was approved by the Institutional Review Board of the Clarion Hospital. A nine-question survey was developed by the first author with the goal of identifying how cancer patients feel about the field of palliative care by asking direct questions with multiple choice answers (Supplementary Fig. S1). Survey questions were designed to be easy to read given the low level of education in the area. Subjects were oncology patients from the cancer center of Clarion Hospital who volunteered to complete the survey. The population studied was a convenience sample in the cancer center, recruited by asking patients in the clinic waiting and infusion rooms whether they would be willing to participate. Results were analyzed based on a total of 65 completed surveys. Data were analyzed by the author and reviewed by a statistician. Arithmetic statistics were utilized.

## Results

**FIG. 1.**
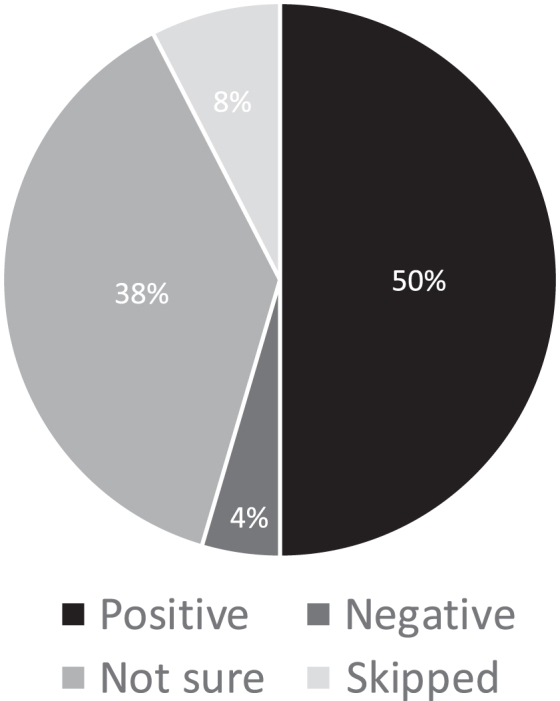
Impression of palliative and hospice care.

In this study, subjects were patients receiving care at the Clarion Hospital Cancer Center. Most of the patients were elderly Caucasian individuals who live in Clarion County, Pennsylvania, or surrounding rural areas. Sixty-five of 66 patients approached agreed to participate. In our convenience sample of 65 patients, 54% of respondents had heard of palliative and hospice care. Fifty percent of those who had heard of the field felt positively toward this field of medicine (Fig. 1).

**FIG. 2.**
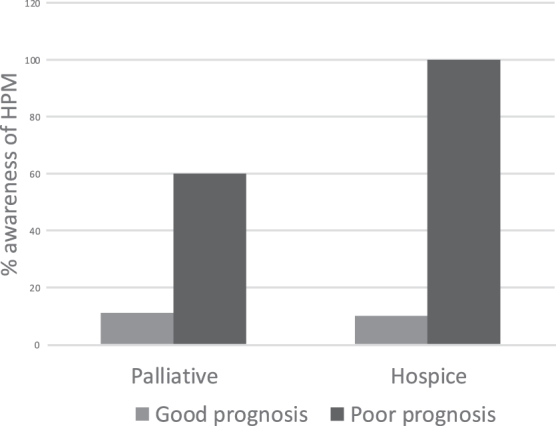
Association of prognosis with awareness of palliative and hospice care. HPM, hospice and palliative medicine.

Patients were asked about their prognosis. Twenty-three percent of patients could not recall being told their prognosis. Approximately two-thirds of patients perceived an overall positive prognosis. For those who self-reported positive prognosis, 11% of the patients had heard of palliative care and 10% had heard of hospice. For patients who self-reported negative prognosis, 100% had heard of hospice care and 60% had heard of palliative care (Fig. 2) despite Clarion Hospital not having a dedicated palliative care or hospice service.

**FIG. 3.**
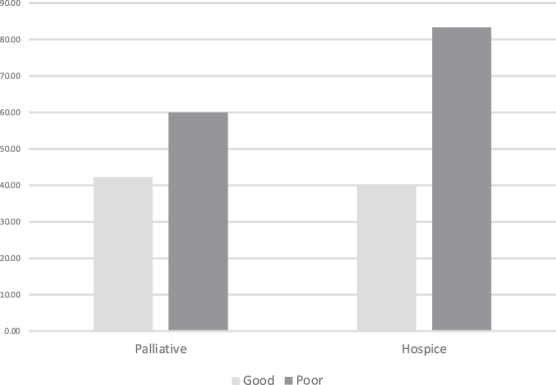
Association of prognosis with perceived needs of palliative and hospice care.

We also asked patients about their future needs for palliative and/or hospice care after explanation of the services. Of patients who understood their cancer had poor prognosis, 60% and 83% believed they would benefit from palliative care and hospice, respectively (Fig. 3). As the patient's self-reported prognosis worsened, their self-perceived need for HPM increased (Fig. 3).

## Discussion

Although awareness of HPM has been increasing in large urban academic centers,^9^ there are currently only a limited number of studies done in the rural setting such as Clarion County, Pennsylvania, which is predominately Caucasian, elderly, and less-educated. As a recent study had shown, awareness of palliative care is significantly lower in rural and micropolitan areas.^7^ However, our data suggest that although rural areas might be limited by resources, there have been improvements in awareness and perception of HPM. Other rural community hospitals in Pennsylvania had revealed similar improvement of awareness.^10^ In addition, more than half of the patients at the Clarion Hospital Cancer Center had positive impressions of HPM, many through their relatives.

In our survey, we further stratified patients' awareness of the field based on their perceived prognosis: good or poor. Their awareness of HPM correlated with the seriousness of their prognosis; good prognosis was associated with less awareness of the field, whereas self-reported poor prognosis had a much higher awareness. It is possible this is due to targeted discussion of these services with those who have more serious illness or that they were better at recalling these discussions. Another explanation can be that, since our cancer center does not have a dedicated HPM service, our patients rely heavily on community hospice services, resulting in delayed introduction until their cancers have progressed to the late stages. However, it is reassuring that at least patients with poor prognosis were more likely to have been introduced to hospice care than the general sample.

This small study has limitations, including a small sample size of only 65 patients and possible selection bias in the participation of patients. Since this study was done in only the Clarion Hospital Cancer Center, it might not be applicable to other bigger institutions. In addition, the survey provided to patients was developed by the author and was a nonvalidated instrument. Future studies should address these limitations. Additional study should focus as well on generalizing studies to multiple centers in different settings.

Overall, our study utilizes an author-developed survey in a small rural community hospital in Pennsylvania. Our data suggest improved awareness and impression of the field of HPM in a rural community cancer center. We hope that the field of HPM will continue to expand in rural areas where advanced subspecialties and technologies are not currently accessible yet remain necessary.

## Supplementary Material

Supplemental data

## Abbreviation Used

HPM: hospice and palliative medicine
