# Supplementary material for: Survey Study of Awareness and Perception of Palliative and Hospice Care in a Cancer Center in Rural Pennsylvania
Source: Palliat Med Rep. 2022 Feb 21;3(1):17–20. doi: 10.1089/pmr.2020.0110 (PMC8900202; doi:10.1089/pmr.2020.0110)
Supplement: Supplemental data [file Suppl_FigureS1.pdf]

Supplemental figure

Study survey used for assessment of palliative and hospice care.

Circle the choice that applies to you:

1. Are you a:

   Patient   Family Member   Non-related caregiver
2. What is the prognosis of the cancer that the oncologist has given?
   - Excellent: no effect on life years
   - Good: decreased life expectancy but will not affect your activities of daily living in the long term
   - Fair: will affect your life but manageable
   - Poor: will affect you for the rest of your life, you will need major changes to your living
   - Very poor: life limiting, there is a time limit of _______ months/years (circle one), hospice care has been discussed with you/your family
   - Unsure: the oncologist has not mentioned this to me
3. What is the most important thing you/your family want help with?
   - Side effects from my chemotherapy
   - Pain from my illness
   - Worry about my life span
   - Worry about my family
   - Others: ____
4. Have you heard about palliative/hospice care? CIRCLE ONE: YES / NO
5. What's your impression of palliative/hospice care and why?
   - Positive, because ____
   - Negative, because ____
   - Not sure, never heard of them.

Palliative care is a branch of medicine that helps patients and their family members cope with any serious diagnosis or illnesses, including cancer. Palliative care can be initiated regardless of life expectancy. Some prefer to view this field as supportive care that patients could get help from. In your case, it could be help with pain due to cancer or any other symptoms due to cancer, or even help with activities of daily living (i.e. waking up too frequently at night).

6. Do you think you will benefit from palliative care? YES / NO
7. What do you think you will gain most from palliative care? CIRCLE ONE
   - Medications
   - Medical equipment
   - Comfort of my home
   - Ability to continue what I enjoy doing
   - Other: ___________________

Hospice care is usually initiated when cancer is severely limiting the patient's life, usually to 6 months or so. Hospice care can be initiated in either a hospice facility or home hospice where care is provided with visiting hospice nurses/physicians.

8. Do you think you will benefit from hospice care? CIRCLE ONE: YES / NO
9. There are hospice physicians and nurses available here in the Clarion Hospital. What kind of help would need from them?
   - Relieving symptoms (pain, nausea, vomiting etc)
   - Talk about my wishes
   - Getting support for my family before or after my death

Patients under palliative/hospice care will still be able to have acute inpatient care in hospitals and in nursing homes. Also included in the care are medical equipment and supplies such as hospital bed, dressings, oxygen, breathing treatment, pain medications etc.
